# Do coagulation or fibrinolysis reflect the disease condition in patients with soft tissue sarcoma?

**DOI:** 10.1186/s12885-022-10106-4

**Published:** 2022-10-18

**Authors:** Kunihiro Asanuma, Tomoki Nakamura, Takayuki Okamoto, Tomohito Hagi, Kouji Kita, Koichi Nakamura, Yumi Matsuyama, Keisuke Yoshida, Yumiko Asanuma, Akihiro Sudo

**Affiliations:** 1 Department of Orthopedic Surgery, Mie University School of Medicine, 2-174 Edobashi, 514-8507 Tsu City, Mie Japan; 2 Department of Pharmacology, Faculty of Medicine, Shimane University, Izumo, Shimane, Japan

**Keywords:** Soft tissue sarcoma, Metastasis, Prognosis, Coagulation, Fibrinolysis, D-dimer, plasmin-α2 plasmin inhibitor complex, Soluble fibrin, And thrombin-antithrombin III complex

## Abstract

**Background:**

Coagulation and fibrinolysis are distinct processes that are highly correlated. Cells control coagulation and fibrinolysis by expression of tissue factor and urokinase-type plasminogen activator receptor on their surface. Tumor cells express these proteins, adjust their microenvironment and induce tumor exacerbation. We hypothesized that the expression of plasma markers for coagulation and fibrinolysis in patients with soft tissue sarcomas (STSs) was dependent on the level of tumor malignancy. To elucidate which markers are predictive of recurrence, metastasis and prognosis, coagulation or fibrinolysis, we analyzed the correlation between plasma levels of thrombin-antithrombin III complex (TAT), soluble fibrin (SF), plasmin-α2 plasmin inhibitor complex (PIC), D-dimer (DD) and clinical parameters in patients with STSs.

**Methods:**

TAT, SF, PIC or DD were measured in pre-treatment blood samples from 64 patients with primary STSs and analyzed with clinicopathological parameters, and 5-year recurrence free survival (RFS), 5-year metastasis free survival (MFS) and 5-year overall survival (OS) were evaluated.

**Results:**

The metastasis group had significantly higher DD (p = 0.0394), PIC (p = 0.00532) and SF (p = 0.00249) concentrations than the group without metastasis. The group that died of disease showed significantly higher DD (p = 0.00105), PIC (p = 0.000542), SF (p = 0.000126) and TAT (p = 0.0373) than surviving patients. By dividing the patients into low and high groups, the group with high DD, PIC, SF and TAT showed significantly lower 5-year MFS and 5-year OS than the corresponding low group. Furthermore, in multivariate COX proportional hazard analysis of continuous variables for 5-year MFS, only PIC was found to be a significant factor (HR: 2.14).

**Conclusion:**

Fibrinolysis was better than coagulation at reflecting the disease condition of patients with STS. Notably, PIC levels ≥ 1.1 can not only predict the risk of metastasis and poor prognosis, but also increasing PIC levels correspond to further increases in risks of metastasis and poor prognosis.

**Supplementary information:**

The online version contains supplementary material available at 10.1186/s12885-022-10106-4.

## Background

Coagulation and fibrinolysis are distinct processes that are highly correlated. Fibrin production and degradation are important phenomena in vascular disease and injuries. The expression of tissue factor (TF), the primary initiator of extrinsic coagulation cascade, leads to the generation of thrombin and fibrin. Similarly, expression of urokinase-type plasminogen activator receptor (uPAR) on the cell surface results in the formation of a complex with urokinase-type plasminogen activator (uPA), converts plasminogen into plasmin, and leads to fibrin degradation. Cells can control their microenvironment by adjusting the expression of TF and uPAR. In particular, tumor cells overexpress these factors and alter their microenvironment, a process that is deeply involved in tumor exacerbation.

TF expression is typically observed in endothelium, leukocytes and monocytes. Many tumor cells express TF, including lung cancer, pancreatic cancer, prostate cancer, laryngeal carcinoma, glioma, ovarian cancer, breast cancer and osteosarcoma cells [1–8]. Additionally, circulating TF-positive extracellular vesicles have been observed in lung cancer, breast cancer, pancreatic cancer and leukemia. [9–12]. These various sources of TF expression are powerful triggers for thrombin generation. TF expression is associated with poor prognosis in several malignant tumors [7, 13, 14]. TF is a potential molecular target for cancer therapy and TF inhibitors have been shown to suppress tumor exacerbation in vivo [15]. Following TF expression, the generated thrombin stimulates tumor cell adhesion to platelets, endothelial cells, extracellular matrix proteins, as well as tumor cell mitogenesis and invasion [16–19]. Furthermore, thrombin can induce secretion of various cytokines and proteases. Notably, thrombin stimulates VEGF expression in tumor and endothelial cells, and is involved in angiogenesis [20–23]. The biological activity of thrombin is mediated by the thrombin receptor protease-activated receptor-1 (PAR-1) [24], and PAR-1 is a promising molecular target for cancer treatment [25]. Thus, activation of coagulation leads to exacerbation of malignant tumors.

In contrast, uPAR is usually expressed in endothelial cells [26], fibroblasts [27, 28], neutrophils and monocytes [29]. Moreover, a variety of malignant cells expresses uPAR, such as breast cancer, gastric cancer, lung cancer and sarcoma cells [30–35]. uPAR-expressing tumor cells can activate oncogenic pathways such as MAPK, RTK, ERK2 and FAK [36–38]. HIF-1 increase in cancer cells lead to upregulation of uPA/uPAR [39]. Plasmin generated by uPA causes a variety of proteolytic events, including fibrin degradation. Plasmin activates pro-MMPs and cleaves extracellular matrix components [40]. Overexpression of uPAR in breast cancer enhances tumor invasion, growth and metastasis [41]. Furthermore, uPAR expression is related to prognosis in oral cancer [42]. Given this, activation of fibrinolysis leads to exacerbation of malignant tumors.

Coagulation and fibrinolytic state can be assessed using several markers, including thrombin-antithrombin III complex (TAT), soluble fibrin (SF), prothrombin fragment 1 + 2, plasmin-α2 plasmin inhibitor complex (PIC), D-dimer (DD) and fibrin degradation products (FDP). Of these markers, TAT, SF, PIC and DD were evaluated in this study. After thrombin generation by activated coagulation cascade, a proportion of thrombin forms a complex with antithrombin III (TAT) [43]. Fibrin monomer generated by thrombin forms a complex with fibrinogen (SF) [44]. On the other hand, Fibrinolytic state can be assessed by fibrinolysis markers, such as PIC or DD. After plasmin is generated from plasminogen, a proportion of the plasmin forms a complex with α2 plasmin inhibitor (PIC) [45]. DD is a degradation product of cross-linked D fragments of fibrin, generated from proteolysis by plasmin [46]. DD levels are indicative of plasmin-induced fibrinolysis after fibrin formation [47].

STSs are categorized as rare cancers, and the relationship between STS and coagulation and fibrinolysis is sparsely reported. We previously reported increased PIC levels were the most suitable for the detection of STS from soft tissue tumors [48]. Based on the findings described above, we hypothesized that plasma markers of coagulation and fibrinolysis in patients with STS is affected by sarcoma tissues and is dependent on its malignancy. We analyzed the correlation between plasma levels of TAT, SF, PIC or DD and clinical parameters in patients with STS to elucidate which markers predict the recurrence, metastasis and prognosis of patients with STS.

## Methods

Patients.

A total of 64 patients with primary STSs who visited Mie University Hospital from 2012 to 2014 were enrolled in this study. Patients who had local recurrence or who were referred for additional resection after inadequate resection in a previous hospital, or had trauma, surgical treatment, thrombosis and disseminated intravascular coagulation (DIC) were excluded from this study. Histopathological diagnosis and the histological grade were verified based on the French Federation of Cancer Centers Sarcoma group system (FNCLCC) by independent pathologists. Patients were classified from stage I to IV according to the 7th edition of the American Joint Committee on Cancer (AJCC) classification of soft tissue sarcomas. Blood samples from all patients were collected in sodium citrate before biopsy or treatment.

The levels of TAT, SF, PIC or DD in plasma were quantitatively measured using a chemiluminescent enzyme immunoassay or latex photometric immunoassay. Written, informed consent was obtained from each patient. For patients below 19 years of age, informed consent was obtained from their parents or legal guardian. This study was approved by the Ethics Committee of the Mie University Graduate School of Medicine (approval number: 1310). All procedures performed in studies involving human participants were in accordance with the ethical standards of the Ethics Committee of Mie University and with the Helsinki declaration of 1975.

### Statistical analysis

Clinicopathological analysis was performed to compare the plasma levels of TAT, SF, PIC or DD to various clinical parameters using the Mann-Whitney test or Kruskal Wallis test for quantitative data and Fisher’s exact test for qualitative data. To evaluate the threshold for detecting recurrence, metastasis or mortality due to disease, receiver operating characteristic (ROC) curves were generated. ROC curves were created by plotting the sensitivity on the y-axis and the false positive rate (1-specificity) on the x-axis. To measure the effectiveness of TAT, SF, PIC or DD levels as a marker for recurrence, metastasis or mortality due to disease, the area under the curve (AUC) was assessed. Local recurrence-free survival (RFS) was defined as the time from initial treatment to the date of clinically documented local recurrence. Metastasis-free survival (MFS) was defined as the time from initial treatment to the date of clinically documented distant metastasis. Overall survival (OS) was defined as the time from initial treatment to the date of mortality attributed to the neoplasm. Kaplan-Meier survival plots and log-rank tests were used to assess the differences in time to local recurrence, distant metastasis or overall survival. To adjust the imbalance in prognostic factors among patients, Cox proportional hazard analysis was used. p < 0.05 was considered statistically significant. The EZR software program was used for statistical analyses [49].

## Results

### Patient and tumor characteristics

A total of 64 patients with primary STSs visiting Mie University Hospital from 2012 to 2014 were included in this study. The average age of the patients was 63 years (range: 17–89 years), and the average tumor size was 12.8 cm (range: 3.5–31 cm). Histopathological diagnosis was as follows: 28 liposarcomas (17 well-differentiated liposarcomas, 6 dedifferentiated liposarcomas, and 5 myxoid liposarcomas), 10 undifferentiated pleomorphic sarcomas, 9 myxofibrosarcomas, 4 leiomyosarcomas, 3 synovial sarcomas, 3 malignant peripheral nerve sheath tumors, and 7 others in STS. Patients with STS received wide resection (44 patients), marginal resection (17 patients) and ion beam radiotherapy (3 patients). The average follow-up on patients with STS was 43.4 months (range: 0.6–75.6 months).

## Characteristics of soft tissue sarcoma

**Fig. 1:**
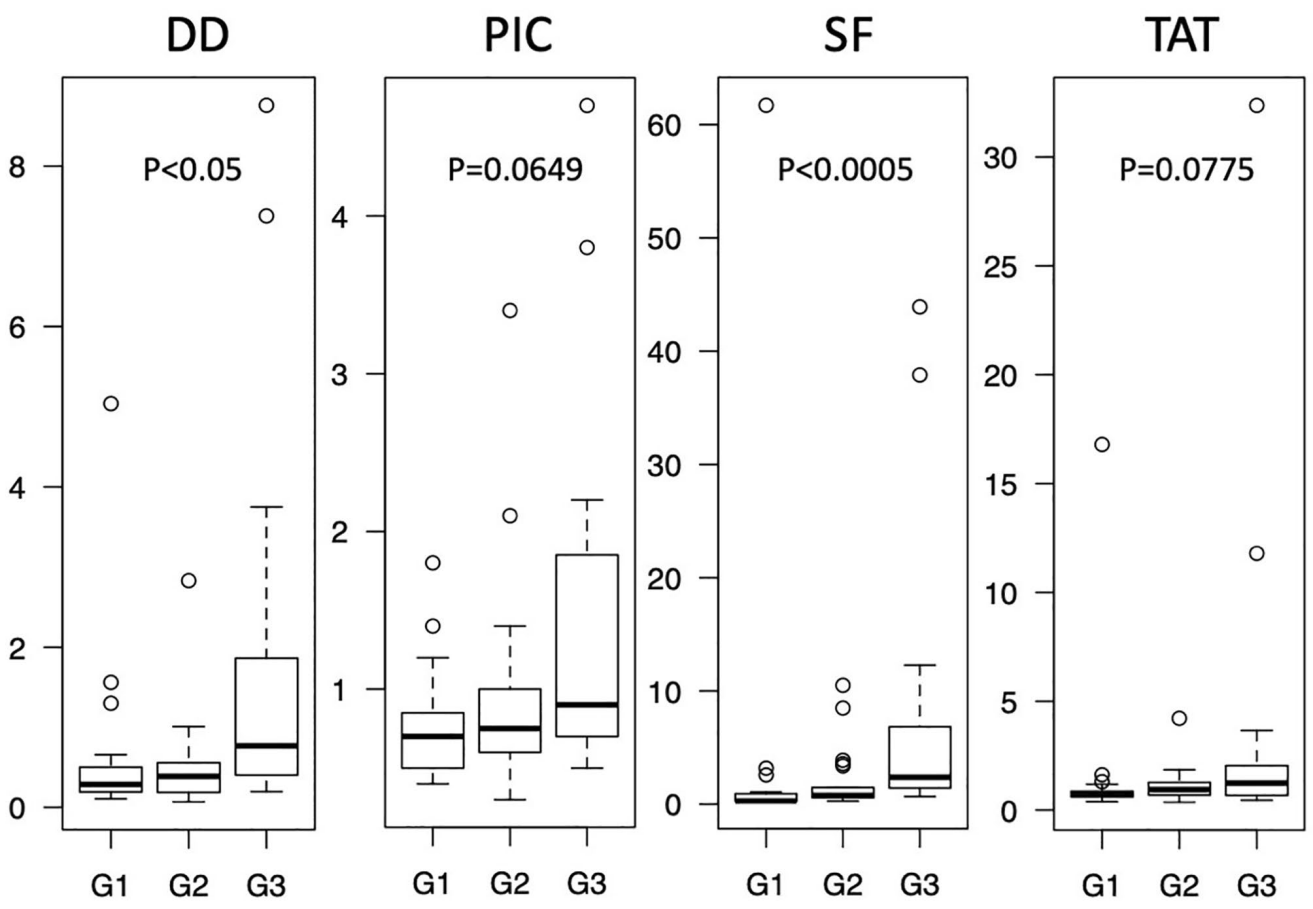
BOX plot of plasma DD, PIC, SF and TAT levels according to FNCLCC grade. G1, G2 and G3 correspond to FNCLCC grades 1, 2 and 3. Statistical analysis was performed by the Kruskal-Wallis test

**Fig. 2:**
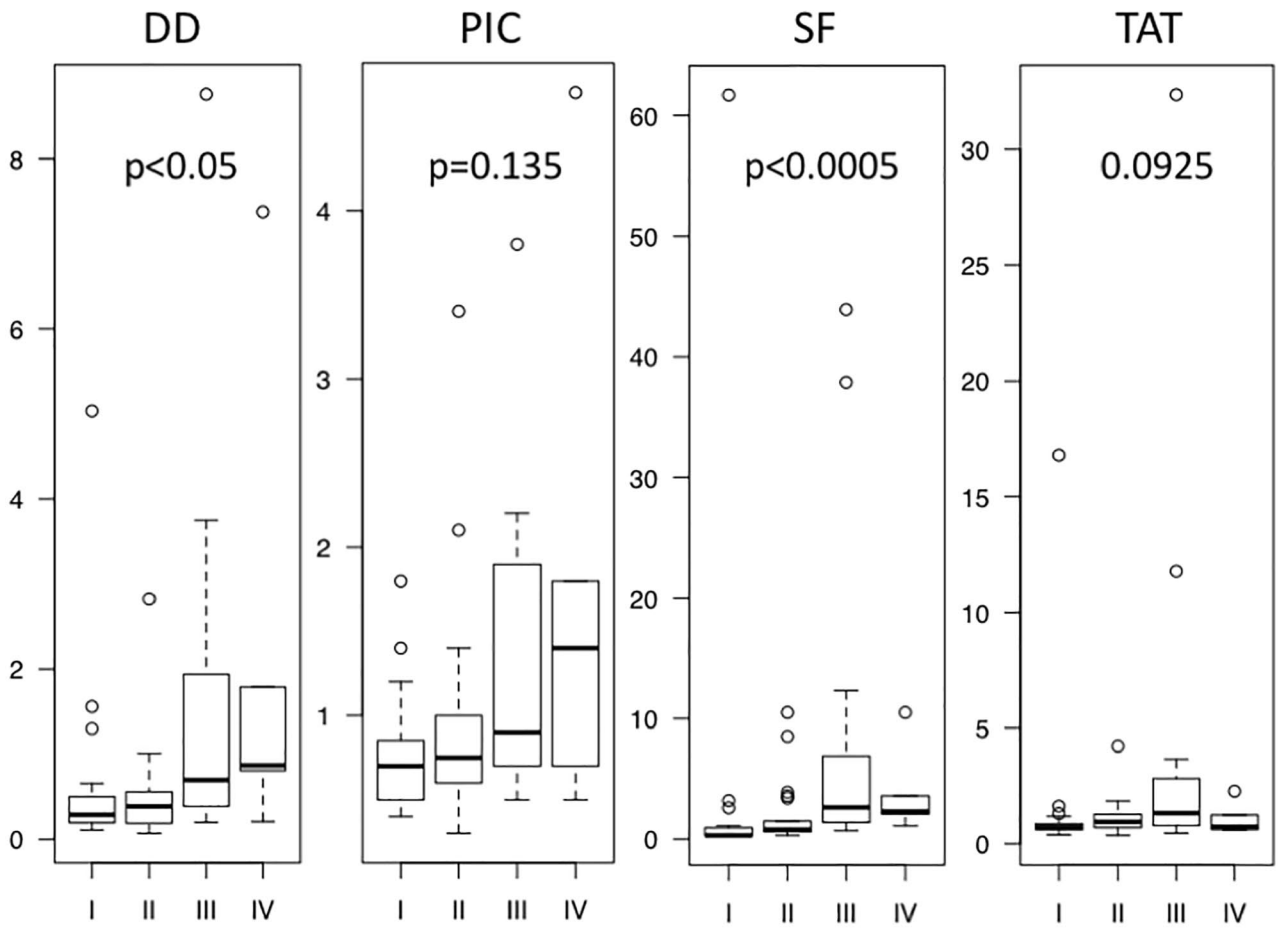
BOX plot of plasma DD, PIC, SF and TAT levels according to AJCC stage. I, II, III and IV correspond to AJCC stages I, II, III and IV. Statistical analysis was performed by the Kruskal-Wallis test

Plasma STS, DD, PIC, SF and TAT values were compared according to sex, age, tumor size, location, tumor depth, FNCLCC grade, AJCC stage and treatment. Patients ≥ 60 years of age showed significantly higher DD, PIC, SF and TAT than those under 60. Patients with tumors ≥ 10 cm in size showed significantly higher DD than those with tumors < 10 cm. In evaluating FNCLCC grade, DD and SF were significantly different depending on FNCLCC grade. However, the average DD, SF and TAT levels of grade 1 were higher than in grade 2 (Table 1; Fig. 1). DD and SF were found to significantly vary according to AJCC stage. However, the lowest levels of DD and SF were observed in stage 2. PIC levels were observed to increase according to stage, despite a lack of statistical significance (Table 1; Fig. 2).

**Table 1:** Plasma levels of DD, PIC, SF and TAT according to various characteristics of patients with STS.

Characteristics of STS		N (64)	DD AVE (SD)	PIC AVE (SD)	SF AVE (SD)	TAT AVE (SD)
Sex	Male	33	1.086 (1.639)	1.000 (0.910)	2.990 (6.804)	1.090 (0.813)
	Female	31	0.904 (1.579)	1.093 (0.689)	5.783 (13.11)	2.988 (6.432)
Age (years old)	< 60	27	*0.616 (1.403)	*0.781 (0.802)	*1.500 (2.281)	*1.002 (0.789)
	≥ 60	37	*1.276 (1.694)	*1.237 (0.762)	*6.418 (13.18)	*2.744 (5.908)
Tumor size	< 10	29	*0.792 (1.434)	1.062 (1.063)	2.217 (3.053)	1.112 (0.859)
(cm)	≥ 10	35	*1.169 (1.728)	1.031 (0.521)	6.105 (13.57)	2.753 (6.079)
Location	Extremity	35	0.946 (1.617)	0.934 (0.600)	4.065 (9.540)	1.446 (1.989)
	Trunk	29	1.060 (1.605)	1.179 (0.994)	4.679 (11.44)	2.689 (6.439)
Tumor depth	Superficial	20	0.907 (1.654)	1.235 (1.066)	7.455 (16.04)	2.409 (4.192)
	Deep	44	1.039 (1.593)	0.959 (0.652)	2.929 (6.096)	1.827 (4.777)
FNCLCC	Grade 1	19	^**#**^ **0.673 (1.124)**	0.784 (0.365)	^**#**^ **3.957 (14.00)**	1.631 (3.683)
	Grade 2	22	^**#**^ **0.534 (0.587)**	0.940 (0.681)	^**#**^ **1.940 (2.683)**	1.124 (0.816)
	Grade 3	23	^**#**^ **1.710 (2.266)**	1.360 (1.065)	^**#**^ **6.960 (11.28)**	3.168 (6.779)
AJCC stage	I	19	^**#**^ **0.673 (1.124)**	0.784 (0.365)	^**#**^ **3.957 (14.00)**	1.631 (3.683)
	II	22	^**#**^ **0.534 (0.587)**	0.940 (0.681)	^**#**^ **1.940 (2.683)**	1.124 (0.816)
	III	18	^**#**^ **1.571 (2.124)**	1.233 (0.845)	^**#**^ **7.805 (12.56)**	3.744 (7.598)
	IV	5	^**#**^ **2.212 (2.943)**	1.820 (1.693)	^**#**^ **3.920 (3.784)**	1.096 (0.713)
Treatment	Wide resection	44	1.184 (1.767)	1.181 (0.915)	^**#**^ **4.581 (8.657)**	2.181 (4.991)
	Marginal resection	17	0.650 (1.165)	0.776 (0.366)	^**#**^ **4.347 (14.80)**	1.717 (3.895)
	Ion beam radiotherapy:	3	0.243 (0.198)	0.566 (0.152)	^**#**^ **0.833 (0.611)**	1.140 (0.680)
Chemotherapy	-	48	1.115 (1.759)	1.162 (0.894)	4.466 (10.81)	2.286 (5.250)
	+	16	0.648 (0.942)	0.693 (0.211)	3.975 (9.206)	1.178 (0.807)
Radiotherapy	-	52	0.775 (0.955)	0.998 (0.694)	3.990 (10.34)	2.134 (5.050)
	+	12	1.961 (3.032)	1.250 (1.194)	5.875 (10.76)	1.468 (1.162)

Average (AVE) values and standard deviation (SD) of DD, PIC, SF and TAT are shown according to each listed parameter. * and ^#^ indicate significant differences using the Mann-Whitney test (p < 0.05) and the Kruskal-Wallis test (P < 0.05), respectively

## DD, PIC, SF and TAT levels in recurrence, metastasis and DOD in STS

**Fig. 3:**
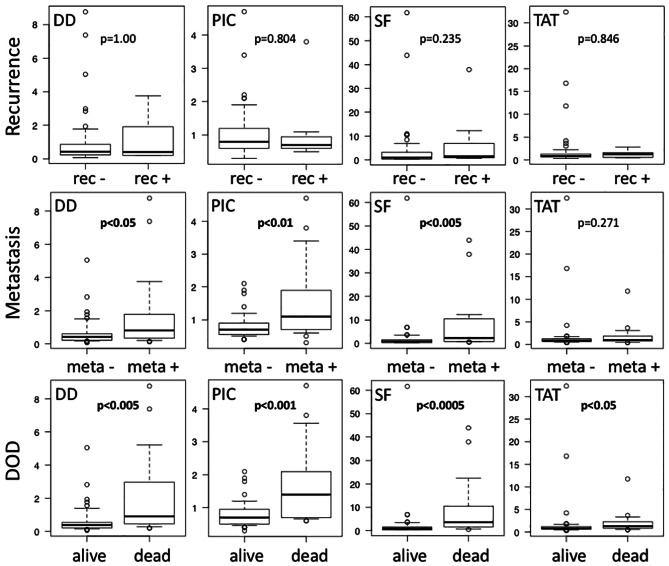
BOX plot of plasma DD, PIC, SF and TAT levels according to clinical outcome. Rec: recurrence, meta: metastasis, DOD: died of disease. Statistical analysis was performed by the Mann-Whitney test (p < 0.05)

During the period of this study, 7 patients developed recurrence, 21 patients developed metastasis (metastasis group), and 17 patients died of disease (DOD group). No significant differences were observed in DD, PIC, SF and TAT between patients with and without recurrence. The metastasis group had significantly higher plasma DD (p = 0.0394), PIC (p = 0.00532) and SF (p = 0.00249) levels than the group without metastasis. The DOD group showed significantly higher DD (p = 0.00105), PIC (p = 0.000542), SF (p = 0.000126) and TAT (p = 0.0373) compared to the surviving patients (Fig. 3).

## COX proportional hazard analysis in STS using continuous variables

Univariate COX proportional hazard analysis was performed using DD, PIC, SF and TAT as continuous variable. For RFS, only stage III/IV was significantly different (HR: 6.20, 95%CI: 1.19–32.1, p = 0.0298). For MFS, DD (HR: 1.26, 95%CI: 1.07–1.50, p = 0.00612), PIC (HR: 2.39, 95%CI: 1.59–3.58, p = 0.000022) and stage III/IV (HR: 3.34, 95%CI: 1.40–7.96, p = 0.006494) showed significant differences. For OS, DD (HR: 1.33, 95%CI: 1.11–1.58, p = 0.001362), PIC (HR: 1.99, 95%CI: 1.42–2.79, p = 0.000059), SF (HR: 1.02, 95%CI: 1.01–1.05, p = 0.026) and stage III/IV (HR: 5.66, 95%CI: 1.98–16.1, p = 0.001164) demonstrated significant differences (Table 2).

**Table 2:** Univariate COX proportional hazard analysis

	RFS			MFS			OS		
	HR	95%CI	p-value	HR	95%CI	p-value	HR	95%CI	p-value
Male	4.9 × 10^8^	/	0.998	0.78	0.33–1.84	0.573	0.67	0.24–1.86	0.447
Age ≥ 60	0.59	0.13–2.65	0.495	1.45	0.58–3.61	0.423	3.32	0.09–11.7	0.063
Size ≥ 10 cm	0.84	0.18–3.78	0.828	0.98	0.41–2.31	0.965	1.66	0.59–4.67	0.335
Superficial	0.25	0.03–2.11	0.204	0.88	0.35–2.18	0.787	1.11	0.39–3.15	0.830
Trunk	2.49	0.55–11.2	0.233	1.71	0.72–4.05	0.218	2.09	0.75–5.81	0.154
DD	1.11	0.77–1.59	0.562	1.26	1.07–1.50	**0.0061**	1.33	1.11–1.58	**0.0014**
PIC	1.26	0.60–2.65	0.534	2.39	1.59–3.58	**0.00003**	1.99	1.42–2.79	**0.00006**
SF	1.02	0.98–1.07	0.219	1.02	0.99–1.04	0.096	1.02	1.01–1.05	**0.026**
TAT	0.95	0.71–1.27	0.736	0.98	0.89–1.09	0.829	1.01	0.92–1.10	0.855
High grade	0.025	0.0-16.2	0.264	39.3	0.79–1957	0.065	67.2	0.76–51.3	0.087
Stage I, II vs. III, IV	6.20	1.19-32.1-	**0.029**	3.34	1.40–7.96	**0.007**	5.66	1.98–16.1	**0.0011**

Plasma DD, PIC, SF and TAT levels were analyzed as continuous variables

Multivariate COX proportional hazard analysis was used to adjust for the imbalance in prognostic factors among patients. Since only stage III/IV showed a significant difference in RFS, multivariate analysis was not performed. DD, PIC and stage III/IV were adopted for multivariate analysis of MFS, with only PIC exhibiting a significant difference (HR: 2.14, 95% CI: 1.26–3.62, p = 0.00477). DD, PIC, SF and stage III/IV were adopted for multivariate analysis of OS, with only stage III, IV exhibiting a significant difference (HR: 4.54, 95% CI: 1.46-14.0, p = 0.00872). PIC showed a risk of poor prognosis, but there was no significant difference (HR: 1.75, 95%CI: 0.96–3.19, p = 0.0655) (Table 3).

**Table 3:** Multivariate COX proportional hazard analysis

	MFS				OS		
	HR	95%CI	p-value		HR	95%CI	p-value
DD	0.99	0.77–1.27	0.961	DD	0.93	0.65–1.31	0.692
PIC	2.14	1.26–3.62	**0.0048**	PIC	1.75	0.96–3.19	0.065
Stage III, IV	2.38	0.94–6.06	0.067	SF	1.028	0.99–1.06	0.108
				Stage I, II vs. III, IV	4.54	1.46-14.0	**0.0087**

Plasma DD, PIC and SF levels were analyzed as continuous variables

For further prognostic analysis, patients with STS of FNCLCC grade 1 or with distant metastasis at initial diagnosis (stage IV) were excluded. Univariate COX proportional hazard analysis indicated that only SF was significantly different in RFS (HR: 1.08, 95%CI: 1.01–1.16, p = 0.013). For MFS, significant differences were detected in DD (HR: 1.22, 95%CI: 1.00-1.49, p = 0.049), PIC (HR: 2.65, 95%CI: 1.53–4.59, p = 0.00051) and SF (HR: 1.07, 95%CI: 1.03–1.11, p = 0.00054); whereas for OS, significant differences were detected in DD (HR: 1.35, 95%CI: 1.10–1.67, p = 0.0043), PIC (HR: 2.45, 95%CI: 1.47–4.08, p = 0.00057) and SF (HR: 1.07, 95%CI: 1.03–1.11, p = 0.00028) (Supplementary Table 1). DD, PIC and SF were adopted for multivariate analysis. PIC (HR: 2.37, 95%CI: 1.31–4.29, p = 0.004) and SF (HR: 1.06, 95%CI: 1.01–1.12, p = 0.013) exhibited significant differences in MFS, while PIC (HR: 2.13, 95%CI: 1.21–3.72, p = 0.0079) and SF (HR: 1.05, 95%CI: 1.00-1.11, p = 0.025) were significantly different in OS (Supplementary Table 2).

## Determination of threshold by ROC analysis for identifying 5-year DOD

**Fig. 4:**
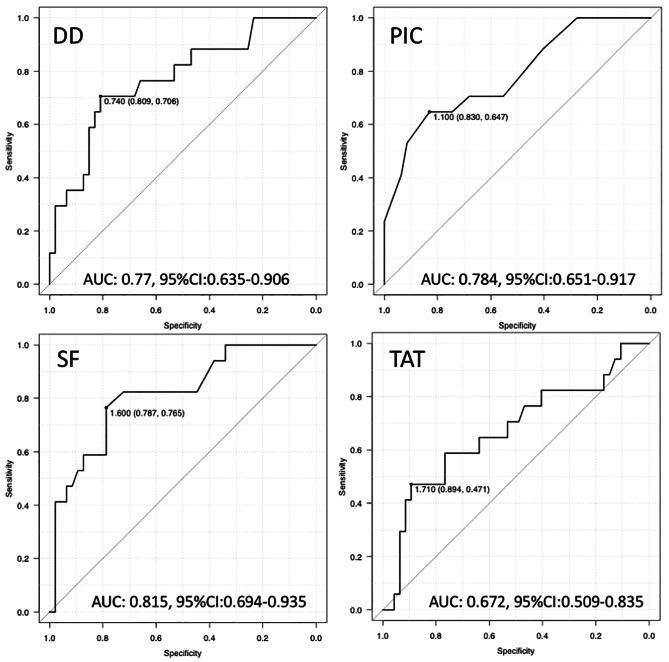
ROC analysis. ROC analysis of DD, PIC, SF and TAT to determine diagnostic accuracy of detecting DOD in sarcoma patients. AUC: area under the curve. Thresholds were determined by the Youden index

ROC analysis was performed and UAC was evaluated to determine the diagnostic accuracy for identifying DOD within 5-years in STS. ROC analysis of DD produced an AUC of 0.77 (95% confidence interval (CI): 0.635–0.906). With a threshold of 0.74, the sensitivity and specificity for identifying 5-year DOD were 70.6% and 80.9%, respectively. ROC analysis of PIC produced an AUC of 0.784 (95% CI: 0.651–0.917). With a threshold of 1.1, the sensitivity and specificity for identifying 5-year DOD were 64.7% and 83.0%, respectively. ROC analysis of SF produced an AUC of 0.815 (95% CI: 0.694–0.935). With a threshold of 1.6, the sensitivity and specificity for identifying 5-year DOD were 76.5% and 78.7%, respectively. ROC analysis of TAT produced an AUC of 0.672 (95% CI: 0.509–0.835). With a threshold of 1.71, the sensitivity and specificity for identifying 5-year DOD were 47.1% and 89.4%, respectively. To divide patients into two groups for further analysis, thresholds of 0.74 (DD), 1.1 (PIC), 1.6 (SF) and 1.71 (TAT) were adopted as the Youden index, and the low and high groups for each threshold were analyzed. (Fig. 4).

## Kaplan-Meier analysis in STS

**Fig. 5:**
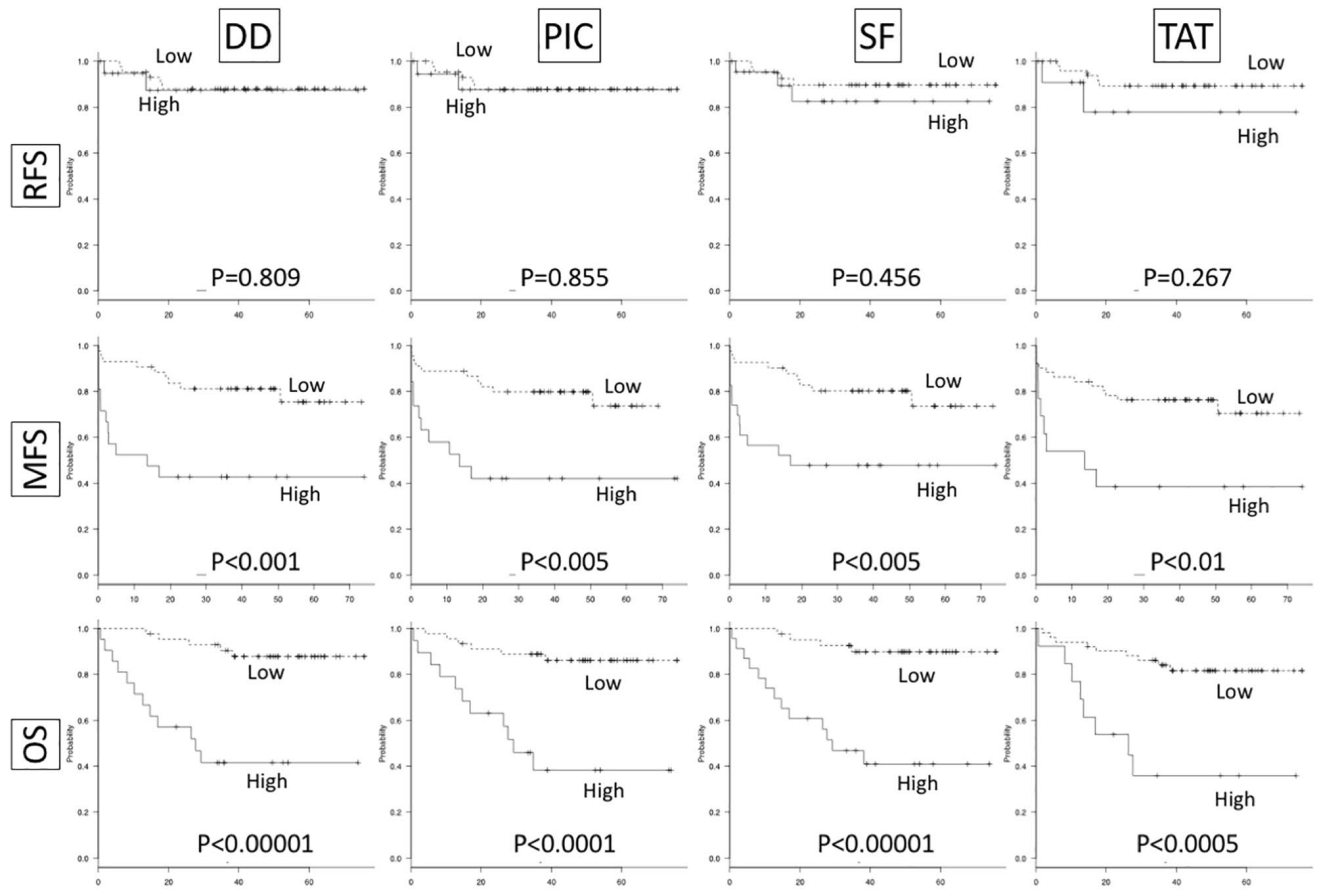
Kaplan-Meier analysis. Kaplan-Meier analysis was performed according to the low and high expression sub-groups for each marker (DD, PIC, SF, and TAT). RFS indicates recurrence-free survival. MFS indicates metastasis-free survival. OS indicates over-all survival. Significance was analyzed using the log-rank test

DD (low < 0.74 ≤ high), PIC (low < 1.1 ≤ high), SF (low < 1.6 ≤ high) and TAT (low < 1.71 ≤ high) were divided into two groups. Five-year recurrence-free survival (RFS), metastasis-free survival (MFS), and over-all survival (OS) between the low- and high-DD, PIC, SF and TAT groups were compared using Kaplan-Meier analysis and the log-rank tests. RFS showed no significant difference in DD (low DD 88.1%, high DD 87.4%, p = 0.809), PIC (low PIC 87.9%, high PIC 87.7%, p = 0.855), SF (low SF 89.8%, high SF 82.6%, p = 0.456) or TAT (low TAT 89.4%, high TAT 77.9%, p = 0.267). MFS showed significant differences in DD (low DD 75.4%, high DD 42.9%, p = 0.000628), PIC (low PIC 73.6%, high PIC 42.1%, p = 0.00164), SF (low SF 73.5%, high SF 47.8%, p = 0.0047) and TAT (low TAT 70.4%, high TAT 38.5%, p = 0.00556). OS and MFS showed significant differences in DD (low DD 87.7%, high DD 41.6%, p = 0.00000601), PIC (low PIC 86.1%, high PIC 38.3%, p = 0.0000516), SF (low SF 89.8%, high TM 41.0%, p = 0.00000925) and TAT (low TM 81.6%, high TM 35.9%, p = 0.000143) (Fig. 5). Thus, high levels of DD, PIC, SF and TAT appear to be important factors involved in metastatic potential and lead to poor prognosis.

## COX proportional hazard analysis of STS with binary DD, PIC, SF and TAT variables

The low and high DD, PIC, SF and TAT groups were analyzed using univariate COX proportional analysis. For RFS, no significant differences were observed between the low and high groups for DD, PIC, SF and TAT. In contrast, analysis of MFS and OS revealed significant differences between the low and high groups for DD, PIC, SF and TAT (Table 4). Significance was mutually nullified by inclusion of high DD, high PIC, high SF and high TAT in multivariate analysis, thus separate analyses were performed with stage III/IV. High DD, high PIC and high SF showed significant differences in MFS and all high groups showed significant difference in OS (Table 5).

**Table 4:** Univariate COX proportional hazard analysis

	RFS			MFS			OS		
	HR	95%CI	p-value	HR	95%CI	p-value		95%CI	p-value
High DD	0.96	0.18–4.99	0.964	3.58	1.48–8.66	**0.0045**	11.4	3.16-41.0	**0.0002**
High PIC	1.03	0.19–5.33	0.969	3.16	1.38–7.93	**0.0071**	8.19	2.57–25.7	**0.0004**
High SF	0.73	0.14–3.80	0.713	2.59	1.08–6.20	**0.032**	8.41	2.35–30.1	**0.0011**
High TAT	1.75	0.33–9.14	0.505	2.71	1.11–6.64	**0.028**	5.80	1.53–12.4	**0.0056**

Plasma DD, PIC, SF and TAT levels were divided into low and high groups by their respective thresholds (Youden index). COX analysis was performed using binary variables

**Table 5:** Multivariate COX proportional hazard analysis

	MFS			OS		
	HR	95%CI	p-value		95%CI	p-value
High DD	3.05	1.2–7.7	**0.019**	11.3	3.16-41.0	**0.0002**
Stage I, II vs. III, IV	2.31	0.91–5.83	0.076	5.66	1.98–16.1	**0.0012**
High PIC	2.90	1.19–7.03	**0.019**	8.19	2.57–25.7	**0.0004**
Stage I, II vs. III, IV	2.66	1.08–6.50	**0.032**	5.66	1.98–16.1	**0.0012**
High SF	2.21	1.08–6.20	**0.032**	8.41	2.35–30.1	**0.0011**
Stage I, II vs. III, IV	2.33	0.87–6.19	0.088	5.66	1.98–16.1	**0.0012**
High TAT	2.71	1.11–6.64	0.111	5.80	1.53–12.40	**0.0056**
Stage I, II vs. III, IV	3.34	1.40–7.96	**0.0065**	5.66	1.98–16.1	**0.0012**

Plasma DD, PIC, SF and TAT levels were analyzed as continuous variables. From Table 3, stage III/IV had significant difference and was adopted this analysis

## Discussion

The intimate relationship between coagulation and fibrinolysis in malignant tumors is a common and well-known phenomena. This study is the first to report on the association between STSs and coagulation and fibrinolysis factors. In this study, plasma levels of DD, PIC, SF and TAT were evaluated to assess the state of coagulation and fibrinolysis in patients with STS. Enhanced coagulation activity or activation of the coagulation cascade originating from TF on normal cells, tumor cells or micro vesicles can be monitored by assessing SF and TAT. In a study examining the relationship between SF and tumors, SF was found to bind lymphocytes and tumor cells, thereby inhibiting both cell adherence and promoting tumor cell resistance to cytotoxicity [50]. High levels of SF in tumor tissues of lung cancer were found to be related to poor prognosis [51]. TAT has proven to be a useful marker for discriminating patients with benign and malignant ovarian tumors [52]. Furthermore, increases in plasma TAT levels appear to be related to tumor spread in lung cancer [53]. Although many tumor cells are known to be highly correlated with coagulation, reports regarding the association between SF, TAT and prognosis are not common.

In contrast, many studies on the relationship between fibrinolysis makers and malignant tumors have been reported. DD is correlated with prognosis in patients with various malignant tumors. High DD levels are associated with poor prognosis in patients with breast cancer, renal cell carcinoma, gastric cancer, lung cancer, bladder cancers, colorectal cancers, gynecological tumors and lymphoma and sarcoma [47, 54–61]. High levels of PIC have been reported to be correlated with poor prognosis in lung cancer [62]. For sarcomas, studies have mainly analyzed D-dimer, reporting that high DD levels are associated with poor prognosis [63–66]. However, there have been no reports evaluating coagulation and fibrinolysis states in patients with STS. Here, we elucidated this relationship by comparing the severity of coagulation and fibrinolysis with the disease condition in sarcoma patients.

In clinical situations, higher stage diagnoses are highly correlated with poor prognosis in patients with STS [67]. Histological grade [68] and stage indicate tumor aggressiveness and spread, information that is indispensable to making decisions about tumor treatment strategies. In considering FNCLCC grades, the levels of DD and SF were found to be significantly different (Fig. 1). Similarly, the levels of DD and SF differed significantly according to AJCC stage (Fig. 2). However, only PIC levels were increased in both higher grade and higher stage, with DD showing a similar, if not significant, pattern. This indicates that PIC, and probably DD, is correlated with malignancy and tumor progression.

In the subsequent analysis, thresholds were adopted to determine the utility of DD, PIC, SF and TAT as predictive markers of prognosis. Thresholds of ≥ 0.74 DD, ≥ 1.1 PIC, ≥ 1.6 SF successfully demonstrated risk of metastasis and poor prognosis. Specifically, DD levels ≥ 0.74 (HR: 3.05) and SF levels ≥ 1.6 (HR: 2.21) can be used as independent predictors of future metastasis (Tables 4 and 5). DD and SF were useful as diagnostic tools only when evaluated using a threshold. In contrast, univariate COX analysis of continuous variables indicated that SF and TAT show weak or no association with metastasis (HR: 1.02, 0.98) and prognosis (HR: 1.02, 1.01) (Table 2). Increases in coagulation markers were not accurately associated with the disease condition. Univariate analysis of fibrinolysis markers indicated that the HR of metastasis (DD: 1.26, PIC: 2.39) and prognosis (DD: 1.33, PIC: 1.99) was higher than with coagulation markers (Table 2). Furthermore, multivariate COX analysis indicted that only PIC exhibited a significant difference in metastasis (HR: 2.14) (Table 3). Additionally, in patients with STS (excluding FNCLCC grade 1 and distant metastasis at initial diagnosis), PIC (MFS, HR: 2.37, p = 0.004, OS, HR: 2.13, p = 0.0079) more effectively and significantly predicted metastasis and poor prognosis than SF (MFS, HR: 1.06, p = 0.013, OS, HR: 1.05, p = 0.025) (Supplementary Tables 1, 2). Thus, fibrinolysis markers were more effective than coagulation markers in reflecting sarcoma malignancy and spread in patients with STS. Especially, PIC levels ≥ 1.1 can be used to predict metastasis risk, and further increases in PIC levels correspond to further increases in metastasis risk.

According to previous sarcoma studies, conversion of plasminogen to plasmin was observed on the sarcoma cell surface [69]. High expression of uPA, uPAR and PAI-1 in tumor tissue, and high levels of serum uPAR were associated with poor prognosis [70]. It was also reported that elevated levels of uPA were observed in leiomyosarcoma, malignant fibrous histiocytoma, higher stage malignancies, sarcomas with necrosis, metastasis or local recurrence. High levels of uPA in tumor tissues were reported to be associated with malignant phenotype [71]. Additionally, synthetic ligands targeting EGFR and uPAR effectively induced sarcoma cell death in vitro and suppressed tumor growth in vivo [72, 73]. These previous reports indicate that the uPA/uPAR system is highly associated with sarcoma exacerbation. Furthermore, inhibitors of PAI-I and uPA have been reported to reduce lung metastasis of osteosarcoma cells [74]^,^[34]. Thus, the uPAR/uPA system appears to be involved in mediating metastatic potential, which is consistent with the findings of the present study. PIC was thought to be a sensitive tool for detecting activation of the uPAR/uPA system in STS and led to successful prediction of metastasis and poor prognosis in patients with STS.

## Conclusion

Serum levels of DD ≥ 0.74, PIC ≥ 1.1, SF ≥ 1.6 and TAT ≥ 1.71 were useful as makers for predicting future metastasis and poor prognosis. Moreover, fibrinolysis was better than coagulation at reflecting the disease condition of patients with STS. Notably, PIC levels ≥ 1.1 can not only predict the risk of metastasis and poor prognosis, but also increasing PIC levels correspond to further increases in risks of metastasis and poor prognosis.

Our study had the following limitations. This study was retrospective and the number of patients was small. Statistical analysis could not be performed by each disease subtype, because STSs are rare entity and including many subtypes. Many studies have needed to analyze STS as a group, rather than by each histological classification. The information of concomitant pharmacological treatments were not included in the statistical analysis.

## Electronic supplementary material

Below is the link to the electronic supplementary material.

Supplementary Material 1

## Data Availability

The datasets used and/or analyzed during the current study are available from the corresponding author on reasonable request.

## List of abbreviations

DD: D-dimer.
PIC: plasmin-α2 plasmin inhibitor complex.
SF: soluble fibrin.
TAT: thrombin-antithrombin III complex.
STS: soft tissue sarcoma.
OR: odds ratio.
RFS: recurrence free survival.
MFS: metastasis free survival.
OS: overall survival.
AUC: the area under the curve.
ROC: receiver operating characteristic.
