# Supplementary material for: Do coagulation or fibrinolysis reflect the disease condition in patients with soft tissue sarcoma?
Source: BMC Cancer. 2022 Oct 18;22:1075. doi: 10.1186/s12885-022-10106-4 (PMC9580209; doi:10.1186/s12885-022-10106-4)
Supplement: Supplementary file 1 — Supplementary Material 1 [file 12885_2022_10106_MOESM1_ESM.docx]

Supplementary Table 1. Univariate COX proportional hazard analysis.

|  | RFS | | | MFS | | | OS | | |
| --- | --- | --- | --- | --- | --- | --- | --- | --- | --- |
|  | HR | 95%CI | p-value | HR | 95%CI | p-value | HR | 95%CI | p-value |
| Male | 1.49x10^9^ | / | 0.999 | 0.54 | 0.19-1.49 | 0.234 | 0.79 | 0.26-2.35 | 0.673 |
| Age ≥ 60 | 0.75 | 0.15-3.73 | 0.728 | 1.83 | 0.63-5.30 | 0.260 | 2.76 | 0.75-10.0 | 0.122 |
| Size ≥ 10 cm | 0.57 | 0.10-3.16 | 0.528 | 0.84 | 0.31-2.25 | 0.729 | 1.33 | 0.44-3.97 | 0.608 |
| Superficial | 0.31 | 0.03-2.73 | 0.297 | 0.82 | 0.28-2.38 | 0.721 | 0.74 | 0.22-2.41 | 0.618 |
| Trunk | 4.29 | 0.78-23.6 | 0.093 | 1.68 | 0.62-4.54 | 0.301 | 2.36 | 0.79-7.05 | 0.122 |
| DD | 1.20 | 0.85-1.71 | 0.289 | 1.22 | 1.00-1.49 | **0.049** | 1.35 | 1.10-1.67 | **0.0043** |
| PIC | 1.66 | 0.69-3.94 | 0.251 | 2.65 | 1.53-4.59 | **0.00051** | 2.45 | 1.47-4.08 | **0.00057** |
| SF | 1.08 | 1.01-1.16 | **0.013** | 1.07 | 1.03-1.11 | **0.00054** | 1.07 | 1.03-1.11 | **0.00028** |
| TAT | 0.95 | 0.73-1.24 | 0.760 | 0.99 | 0.90-1.09 | 0.892 | 1.00 | 0.92-1.10 | 0.856 |

Patients with STS of FNCLCC grade 1 or with distant metastasis at initial diagnosis (stage IV) were excluded. Plasma DD, PIC, SF and TAT levels were analyzed as continuous variables.

Supplementary Table 2. Multivariate COX proportional hazard analysis.

|  | MFS | | |  | OS | | |
| --- | --- | --- | --- | --- | --- | --- | --- |
|  | HR | 95%CI | p-value |  | HR | 95%CI | p-value |
| DD | 0.96 | 0.70-1.31 | 0.806 | DD | 1.11 | 0.82-1.52 | 0.692 |
| PIC | 2.37 | 1.31-4.29 | **0.004** | PIC | 2.13 | 1.21-3.72 | **0.0079** |
| SF | 1.06 | 1.01-1.12 | **0.013** | SF | 1.05 | 1.00-1.11 | **0.025** |

Patients with STS of FNCLCC grade 1 or with distant metastasis at initial diagnosis (stage IV) were excluded. Plasma DD, PIC and SF levels were analyzed as continuous variables.
